# Signaling of Macrophages that Contours the Tumor Microenvironment for Promoting Cancer Development

**DOI:** 10.3390/cells9040919

**Published:** 2020-04-09

**Authors:** Justin K. Messex, Crystal J. Byrd, Geou-Yarh Liou

**Affiliations:** 1 Center for Cancer Research and Therapeutic Development, Clark Atlanta University, Atlanta, GA 30314, USA; jmessex@cau.edu (J.K.M.); crystal.byrd@students.cau.edu (C.J.B.); 2 Department of Biological Sciences, Clark Atlanta University, Atlanta, GA 30314, USA

**Keywords:** macrophages, polarization, cell signaling, transcription factors, cytokines, cancer initiation, tumor development, metastasis, tumor environment

## Abstract

The immune response is critical in the maintenance of an organism’s health. The immune response can be broken down into two groups. The innate response, which is fast-acting and rids the body of most foreign material before infection occurs, and the adaptive response, a more specific defense against pathogen composed mostly of antibody production and killer cells. Linking the two responses via cytokine and chemokine secretion are macrophages, motile phagocytic cells that ingest and present foreign material playing a role in the innate and adaptive immune response. Although macrophages are necessary for the survival of an organism, studies have also shown macrophages play a more sinister role in the initiation, progression, and metastasis in tumorous cells. In this comprehensive review, we show how macrophages induce such a response through abnormal cellular signaling and creating a cellular microenvironment conducive for tumor growth and metastasis, as well as the future outlook of this field.

## 1. Introduction

Individuals are constantly exposed to pathogens and foreign material through inhalation, cuts, and ingestion, leading to side effects or infection. To combat the harmful material entering the body, we have developed an immune system that consists of two distinguishable parts, innate immunity and adaptive immunity. These two systems rely on one another to rid the body of bacterial, parasitic, and other types of foreign invaders and are capable of fighting off the majority of harmful material that enters our body. The innate response is the front line of the immune system. It is the first to act when foreign material is detected and contains physical, chemical, and cellular defenses, which help localize the foreign pathogen to one area of the body and preventing the spread and movement of the infection. However, the innate response is not always sufficient to control the spread of infection. Once foreign material overpowers the innate response, the second wave of immune cells, which is a part of the adaptive immune response or acquired immune response, is activated. The T cells and B cells of the adaptive immune response are activated by various cells of the innate immune response, which include macrophages. Upon activation, a pathogen-specific response to destroy the foreign material is initiated. Furthermore, memory cells are produced during the adaptive immune response and are deployed when contact with the same material is encountered in the future to elicit a stronger and quicker response.

Macrophages make up a large portion of the innate immunity. These large white blood cells are motile and detect microscopic foreign material and pathogens, which they engulf, thus providing protection before side effects and infection can occur. Initial interaction of host macrophage cells with foreign material and pathogens activates the innate immune response; the nonspecific immune response described earlier. During this response, macrophages are activated once a bacterial outer membrane component, lipopolysaccharide (LPS), has been detected. The active macrophages then phagocytize bacteria or the foreign material. After ingestion, macrophages sort through bacteria or infected cells and display a peptide antigen on their cell surface, which is recognized by T helper cells. Once a T helper cell is activated by encountering the peptide on the antigen-presenting cell, the adaptive immune response is initiated.

In addition to their role in both innate and adaptive immunity, research has shown that macrophages also play a dynamic role in the body by interacting with immune cells and epithelial cells to regulate the cellular environment through secretion of cytokines and chemokines [1,2,3,4]. Furthermore, studies on various cancers have revealed that macrophages participate in tumor initiation and development, especially the M2 subtype of macrophages [5,6,7,8,9,10]. A comprehensive understanding of macrophage polarization/subtypes and their function in cellular signaling will not only advance our current knowledge on these multifaceted macrophages but also shed light on how to target them for a cancer therapy purpose.

## 2. Macrophage Subtypes after Polarization and Signaling that Leads to their Polarization

To identify macrophages, among other types of immune cells, cell surface proteins, including CD14, CD16, CD64, CD68, CD71, and EMR1, have been used as a pan macrophage marker. Depending on the signaling molecules released by macrophages, traditionally, they can be categorized into either M1 subtype macrophages or M2 subtype macrophages. M1 macrophages, which are stimulated by LPS and interferon gamma (IFN-gamma) ligands binding to either toll-like receptor 4 (TLR4) or IFN-gamma receptors. The downstream target genes in M1 macrophages include Nos2, Ciita, and other inflammatory genes with the purpose of clearing the initial infection [11,12]. M1 macrophages are identified by their production of high levels of pro-inflammatory cytokines, strong microbial properties, high levels of reactive nitrogen and oxygen intermediates, and promotion of the Th1 response in the adaptive immune response [13,14,15]. In addition, CD38, GPR18, and FPR2 have been reported as M1 macrophage surface markers, allowing direct identification of M1 macrophages, especially in the heterogeneous tissue environment [16].

M2 macrophages are activated by IL-4 or IL-13 ligands binding to IL-4 alpha or IL-13 alpha 1 receptors and are characterized by their tissue remodeling abilities, involvement in parasite control, phagocytic activity, and promotion of Th2 cells and tumors [17,18]. M2 macrophages activate Arg1, Fizz1, and CD206 genes after transducing a signal primarily via the Janus kinase (JAK) 1/2/3 and phosphatidylinositol 3-kinase (PI3K) pathways [19,20]. Exclusively expressed CD163 and CD206 surface proteins are commonly used for M2 macrophage identification in addition to M2 subtypes. Although polarization of M2 macrophages is a nuanced process leading to an array of various other subtypes, the most notable ones are M2a, M2b, M2c, and M2d, which are categorized based on stimuli and secreted cytokines and chemokines (Table 1). Notably, these various subtypes of M2 macrophages have been linked to tumor progression and metastasis through their interactions with other components of the tumor microenvironment and their ability to suppress immune responses that eliminate cancer cells [21,22,23,24,25].

Each T helper cell expresses a CD4 surface protein that recognizes antigens expressed on the major histocompatibility complex (MHC) class II molecules. Subsequently, multiple responses are induced depending on the cytokines released from cells in the adaptive immune response. For example, IFN-gamma and tumor necrosis factor (TNF) secreted by the Th1 subtype of T helper cells strengthen macrophages’ killing efficiency, while simultaneously causing proliferation of cytotoxic T cells [26]. On the other hand, cytokines IL-4, IL-5, and IL-6 secreted by Th2 subtype of T helper cells stimulate B cell proliferation and antibody class switching [27,28]. Accumulation of these responses leads to an additive immune response, which helps the body fight off foreign invaders.

## 3. Macrophage Subtypes and their Involvement in Cancer Initiation and Development through their Signaling to Re-Shape the Tumor Environment

The cell environment is key to tumor initiation and development. In the absence of an environment that favors tumor cell growth, cancerous cells will not fully develop due to the precision and highly functional immune surveillance system that is capable of killing abnormal proliferating cells. In this section, we summarized the reported mechanisms of macrophages through their cell signaling and downstream gene expressions to promote different stages of cancer development, including tumor initiation, progression, and metastasis

### 3.1. The Cell Signaling and Subsequent Activation of Transcription Factors in Tumor-associated Macrophage (TAM) that Regulate Cancer Initiation

Studies have shown the importance of various protein effects on tumor initiation. Table 2 provides a summary of the most well-known tumor initiators, which are described in this section. Notably, cyclin D1, a regulatory subunit required for cell cycle progression, and c-myc, a proto-oncogene, have been indicated in their roles of tumor progression in various cancers [29]. Furthermore, the signal transducer and activator of transcription 3 (STAT3) signaling pathway, which controls these downstream targets, has also been linked to tumor initiation in hepatocellular carcinoma as well as its activator IL-6 cytokine released by M2 macrophages. 

Hepatocellular carcinoma (HCC) is the most common type of primary liver cancer. It is often developed in individuals with long-term chronic liver inflammation due to hepatitis B or hepatitis C viral infections. Recent studies have shown that higher levels of cytokine IL-6 secreted by M2 macrophages were not only detected in HCC patient serum but also associated with cancer stage and progression [30,31]. Furthermore, increased numbers of monocytes and leukocytes existed in the peripheral blood samples of these HCC patients, indicating these immune cells could be the source of the increased levels of serum IL-6. Through bone marrow transplantation experiments, deletion of IL-6, specifically in monocytes, delayed liver cancer formation in Mdr2^−/−^ transgenic mice [30], suggesting that IL-6 secreted from the monocytes are directly involved in liver cancer formation. Moreover, in the liver homogenates of Mdr2^−/−^ IL-6^−/−^ mouse, decreased activation of transcription factor STAT3 and its downstream targets cyclin D1 and c-myc were detected in comparison to those of Mdr2^−/−^ IL-6^+/+^ mouse. These results indicated that monocytes’ IL-6 increased STAT3 activation, which in turn upregulated cyclin D1 and c-myc for initiating liver cancer formation.

Other studies using pancreatic ductal adenocarcinoma as a model showed a similar pattern for tumor initiation. However, research showed this model uses different transcription factors to target different genes leading to tumor initiation. 

Pancreatic ductal adenocarcinoma (PDAC), the most common type of pancreatic cancer, are derived from pancreatic acini through acinar-to-ductal metaplasia (ADM). Therefore, an increase in ADM events promotes PDAC initiation. Studies demonstrated that cytokines TNF and RANTES released by macrophages transdifferentiated pancreatic acini to a duct-like phenotype through activation of transcription factor NF-κB and its downstream target gene matrix metalloprotease 9 (MMP9) in the acinar cells [32]. Interestingly, the pancreatic acini that harbor activating mutations of Kras, the most common dominant gene mutation in cancer, can generate an inflammatory environment by upregulating the chemoattractant ICAM-1 to recruit inflammatory macrophages, thus initiating PDAC through potentiation of ADM process [33]. 

Other research has focused on signaling pathways, transcription factors, and target genes in the initiation of brain tumors. In brain tumors, macrophages and the resident brain macrophages, known as microglia, were detected in high-grade gliomas [34,35]. Overexpression of human protein kinase B, known as Akt, in Zebrafish neurons, dedifferentiated these cells to be Sox2^+^ multipotent neural stem cells, which turn into brain tumor cells with increased cell proliferation [36]. Moreover, these preneoplastic neurons highly expressed Sdf1b to recruit macrophages and microglia cells, both of which are Cxcr4b^+^, for promoting their own cell growth. Meanwhile, Akt-expressing neurons in Cxcr4b^−/−^ Zebrafish abolished Akt-induced infiltration of macrophages and microglial cells and preneoplastic neuronal growth. This study indicates how the tumor-initiating cells start to alter the cell environment toward a pro-cancer condition through the recruitment of macrophages. Other research has indicated the influence macrophages have on manipulating the cellular microenvironment to make it more conducive for tumor initiation. Macrophages are capable of secreting an array of cytokines which influence the microenvironment, ultimately increasing cell proliferation. 

During the early stage of prostate cancer initiation, which requires normal prostate epithelial cells to gain their cell proliferation ability, the surrounding cells of the prostate epithelium, such as immune cells, stromal cells, etc., can contribute to this onset process by altering the local cell environment. It has been shown that macrophages are capable of elevating cell proliferation of human PZ-HPV-7 normal prostate epithelial cells in a three-dimensional culture setting through macrophage-secreted cytokines, including CCL3, IL-1ra, osteopontin, M-CSF-1, and GDNF [37]. In addition, stimulation with any one of these cytokines activated ERK and Akt signaling, which led to cell proliferation of normal prostate epithelial cells. Macrophages are either derived from circulating monocytes or established during embryonic development [38]. Co-culture of monocytes with immortalized human normal prostate epithelial RWPE-1 cells in matrigel in 3D for over 24 days resulted in the transformation of RWPE-1 with the increased ability of anchorage-independent growth and of tumor development when subcutaneously transplanting in immunodeficient nude mice, thus indicating a key role of monocytes in prostatic cancer initiation [39]. Furthermore, it was also shown that activation of transcription factor STAT3 and its downstream-regulated genes COX-2 and c-myc were increased in the transformed RWPE-1 cells. Downregulation of tumor suppressor genes, including PTEN and p53, was also found in these monocytes-transformed RWPE-1 cells. These results indicated that not only activation of oncogenes, such as COX-2 and c-myc, but also inactivation of tumor suppressor genes, such as PTEN and p53, participated in the process of prostate epithelial cell transformation toward tumorigenic. On the other hand, androgen receptor (AR) on the monocytes was responsible for transforming events in RWPE-1 cells, as described above, through an upregulation of cytokine CCL4. 

### 3.2. The Cell Signaling and Regulated Transcription Factors of TAM that Promote Cancer Progression

**Figure 1:**
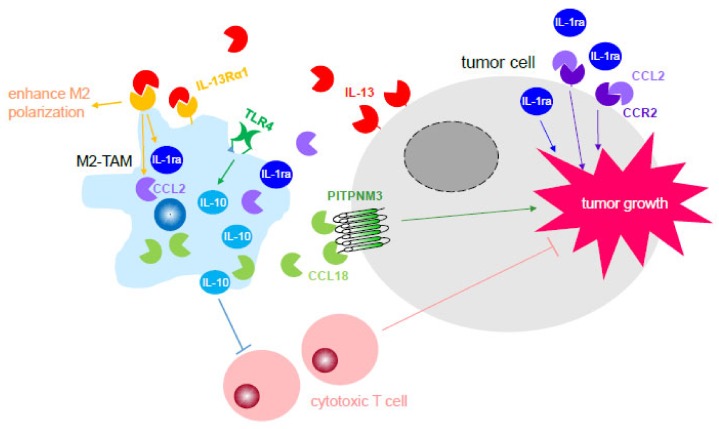
M2-tumor-associated macrophage (M2-TAMs) potentiate tumor growth through their secreted cytokines. Cytokines released by M2-TAMs participated in enhancing tumor growth through an increase in proliferation and inhibition of tumor-killing immune cells, such as cytotoxic T cells. Tumor cells also produce certain cytokines, such as IL-13, that contribute to the polarization of macrophages to become M2-TAMs, thus generating a positive-feedback loop between M2-TAMs and tumor cells during tumor progression. Abbreviation, IL-1ra: interleukin 1 antagonist; IL-10: interleukin-10; TLR4: toll-like receptor 4; PITPNM3: phosphatidylinositol transfer protein membrane-associated 3.

Tumor-associated macrophages contribute to cancer progression through two mechanisms: (1) activation of cell-stimulating growth factors and cytokines through receptor recognition; (2) suppression of antitumor immunity [40,41,42,43] (Figure 1). TAMs are recruited into the tumor by chemoattractants, such as colony-stimulating factors 1 (CSF1) and monocyte chemoattractant protein 1 (MCP-1/CCL2) [42]. The cytokines and their receptors utilized through TAMs during cancer progression include IL-6, IL-12, IL-10, IL-23, TNF, and TLRs. Studies have shown that inhibition of TLR4 signaling in TAMs successfully decreased cytokine production and weakened their tumor-promoting activities [41]. Furthermore, activation of TLR4 signaling on M2-polarized TAMs stimulates IL-10 release, thus promoting cancer progression, especially in the advanced stages of metastatic growth of the tumor. 

#### 3.2.1. Activation of Cell-stimulating Growth Factors and Cytokines through Receptor Recognition to Directly Promote Tumor Progression

Once tumor cell initiation has begun, TAM-mediated secretion of cell signaling molecules results in the upregulation of various transcription factors. The cascade of upregulated transcription factors leads to more aggressive tumor cells by granting them enhanced abilities in cell proliferation, survival, cell migration and invasion, stemness and angiogenesis, therefore, allowing expansion of the tumor cells into new areas of the body.

Pancreatic intraepithelial neoplasia (PanIN) lesions, the precancerous lesions of pancreatic ductal adenocarcinoma, expressed IL-13 leading to an increased population of M2 TAMs found in the local regions [44]. These M2 TAMs were capable of promoting pancreatic fibrosis as well as pancreatic tumorigenesis through their secreted IL-1ra and CCL2, bolstering a key role of TAM in potentiating pancreatic tumor progression. Intriguingly, it has been shown that membrane-associated phosphatidylinositol transfer protein (PITPNM3) allows TAMs-secreted cytokine CCL18 to bind to the cellular membrane causing vascular cell adhesion protein 1 (VCAM-1) upregulation and expression in PDAC cells through transcription factor NF-κB [45]. Activation of this signaling pathway in PDAC cells resulted in a glycolytic phenotype of PDAC cells, which enhances cancer progression. In addition, VCAM-1 induced lactate generation in PDAC cells, causing macrophage polarization in the area. These macrophages demonstrated TAM-like phenotypes establishing a positive feedback loop regulation that favors cancer progression. The regulation of colorectal tumor cells during tumor progression has been associated with NF-κB signaling of TAMs [46,47]. Studies showed that activation of NF-κB in TAMs led to IL-1β secretion from TAMs, which switched the cell signaling pathways between GSK3β and Wnt in colorectal cancer cells. Consequently, turning on Wnt signaling in colorectal cells rendered them highly proliferative through the upregulation of c-Jun and c-Myc [47]. Furthermore, activation of transcription factor STAT1 was essential to increase the levels of IL-1β in TAMs during colorectal tumor progression [46]. 

STAT3 is a critical oncogenic signaling pathway and regulates the M2 subtype macrophages. Accumulating evidence has demonstrated that activation of STAT3 in both tumor cells and TAMs leads to tumorigenesis and tumor progression in several types of cancers, such as glioblastoma, lung cancer, ovarian cancer, and liver cancer [40,43,48,49,50]. Lung cancer invasiveness was increased through TAM-secreted IFN-gamma, which activated JAK/STAT3 and PI3K/Akt signaling in the cancer cells [49]. In addition, treating lung cancer cells with the PI3K/Akt inhibitor LY294002 or the JAK/STAT3 inhibitor AG490 blocked TAM-induced cell migration. In advanced epithelial ovarian cancer, high concentrations of IL-6, IL-10, growth-related oncogene-alpha, and vascular epithelial growth factor (VEGF) were associated with cell proliferation of human ovarian cancer cells [48]. Moreover, it has been shown that knockdown of STAT3 in macrophages that were co-cultured with SKOV3 human ovarian cancer cells inhibited cell proliferation of SKOV3 through the downregulation of IL-6 and IL-10 of macrophages. Intriguingly, STAT3 activation was also detected in SKOV3 cells, which resulted in cellular proliferation in co-culture with macrophages [48]. Other studies have demonstrated the link between cancer progression and abnormal cell signaling mediated by TAM secreted cytokines, including cancer stem cells.

Tumors contain various types of cancer cells, known as tumor heterogeneity. Among these various types, cancer stem cells are critical in cancer progression via their dissemination, which can facilitate tumor angiogenesis and aggressiveness [51]. In addition to the regulation of cancer progression, such as ovarian cancer cells, TAM-secreted IL-6 is also reported to increase the expansion of CD44^+^ stem cells as well as sphere formation of the hepatocellular carcinoma cells [50], thus promoting tumor progression through cancer stem cell growth. Treating human hepatocellular carcinoma cells with tocilizumab, a humanized anti-IL-6 receptor antibody, or knockdown of STAT3 in these cells attenuated the growth of CD44^+^ cancer stem cells that were induced by TAMs. 

One of the main features of tumor cells during tumor progression is their enhanced migration, invasiveness, and angiogenesis through the epithelial–mesenchymal transition (EMT) process. Several lines of evidence have indicated that TAMs are capable of promoting EMT of tumor cells through their secreted factors, including growth factors and cytokines, such as the previously mentioned epidermal growth factor (EGF), and transforming growth factor-β (TGF-β) [52,53,54,55]. Co-culture of M2 macrophages that were polarized from THP1 monocytes with oral cancer or head and neck squamous cancer cells induced the EMT of carcinoma cells through the EGFR pathway and its downstream target ERK in cancer cells [52]. Zinc finger E-box-binding homeobox 1 (ZEB1), a key transcription factor to induce EMT in cancer cells, has been shown to communicate between cancer cells and TAMs for promoting tumor progression [53]. Expression of ZEB1 in macrophages polarized them to become an F4/80^low^ pro-tumor phenotype and contributed to chemoresistance in mice of ovarian cancer. Elevated expression of CCR2 and MMP9 in ZEB1 wildtype F4/80^low^ macrophages provided a positive feedback loop with ZEB1 wildtype cancer cells through cancer cell-expressed CCL2 cytokines [53]. Meanwhile, MMP9 secreted from TAMs containing ZEB1 was shown to upregulate CCL2 expression in ovarian cancer cells. 

The activation of pro-survival signaling pathways in tumor cells, intercellular cross-talk of cancer and stroma cells, and immune microenvironment also plays a role in tumor progression. During tumor progression, cancer cells educate the surrounding stroma cells. Together they tune the cell environment through modifying the extracellular matrices (ECM) to accelerate tumor malignancy and vice versa. For example, versican is an extracellular matrix glycoprotein, which is highly expressed in the early stages of inflammation and neoplastic diseases. In the 4T1 breast cancer mouse model, expression and distribution of versican positively associated with increased peri-tumoral TAM recruitment and breast cancer progression [56]. In addition, increased levels of CCL2, VEGF, and TGF-β1 were correlated to macrophage infiltrated areas with high vascularization and collagen deposits, which indicate further tumor progression. In bladder cancer, collagen has been reported to be secreted by TAMs, leading to cancer growth [57]. The secreted collagen I from TAMs activated the PI3K/Akt signaling pathway through its receptor integrin α2β1 in the bladder cancer cells. The use of the integrin α2β1 inhibitor, E7820, in vitro and in vivo impeded collagen I-induced cell growth of the bladder cancer, indicating the importance of collagen I in bladder cancer progression through its regulation of Akt signaling that can be activated by TAMs. 

#### 3.2.2. Suppression of Anti-tumor Immunity to Indirectly Support Cancer Progression

In addition to directly promoting tumor progression through modulation of tumor cells as described previously, TAMs also inhibit anti-tumor immunity via regulation of tumor-eliminating immune cells, including cytotoxic T cells, natural killer (NK) cells, and regulatory T (Treg) cells. Cytotoxic T cells, also known as cytotoxic T lymphocytes or CD8^+^ T cells, are able to kill neoplastic cells bearing specific antigens through cell lysis. Although NK cells are used by the innate immune response to eradicate abnormal or stressed cells without prior sensitization, they have also been implicated in killing cancer stem cells [58]. Treg cells are a subpopulation of T cells that primarily suppress immune responses through the inhibition of cytotoxic T cell proliferation and secretion of immunosuppressive cytokines. Due to their immune suppressive functions, Treg cells are pivotal to auto-immune disorders as well as cancer development. By impeding cell growth and activation of cytotoxic T cells and NK cells, and expansion of Treg cells result in cancer progression by mitigating the tumor-killing capability of the immune system. 

Several lines of evidence have indicated that TAMs expedite cancer progression by inactivating cytotoxic T cells through direct interactions, while simultaneously decreasing the effects of effector T cells through an increase in Treg cells. TAMs derived from xenografted mice of colon carcinoma or lymphoma induced apoptosis of both CD8^+^ cytotoxic T cells and CD4^+^ helper T cells in vitro through TAMs’ secreted arginase and nitrogen monoxide [59]. Furthermore, the utilization of knockout mice demonstrated that activation of transcription factor STAT1 in TAMs was responsible for the increased arginase- and NO-mediated T cell death. In addition, to eliminate cytotoxic T cells, TAMs also inactivate them through TAM-secreted cytokines, including IL-10, TGFβ, and prostaglandin [60,61,62,63]. So far, the evidence on direct cytokine-mediated cytotoxic T cell suppression by TAMs or other immune cells remains elusive. However, it has been demonstrated with a fluorescently labeled program cell death protein 1 (PD-1) monoclonal antibody conjugated to the PD-1^+^ tumor infiltrating CD8^+^ T cells, PD-1^−^ TAMs are able to transfer fluorescent PD1 monoclonal antibodies from CD8+ T cells to themselves through the Fc gamma receptors of TAMs [64]. These data suggest that TAMs are capable of causing dysfunction in cytotoxic T cells, promoting cancer progression. TAMs have also been implicated in their ability to polarize Treg cells from CD4^+^ help T cells through their secreted TGFβ and IL-10 in epithelial ovarian cancer patients [65], indicating the involvement of TAMs in accelerating cancer progression through weakening anti-tumor immunity.

### 3.3. The Cell Signaling and its Regulated Transcription Factors of TAM that Control Cancer Metastasis

The tumor microenvironment consists of dense areas that present various obstacles, such as hypoxia, neovascularization, immune cell infiltration, etc. As mentioned in the section of macrophage subtypes and polarization, M2 subtype macrophages are mostly found within the tumor. These TAMs release a host of cytokines and chemokines, which are summarized in Table 3, and facilitate cancer metastasis. 

**Figure 2:**
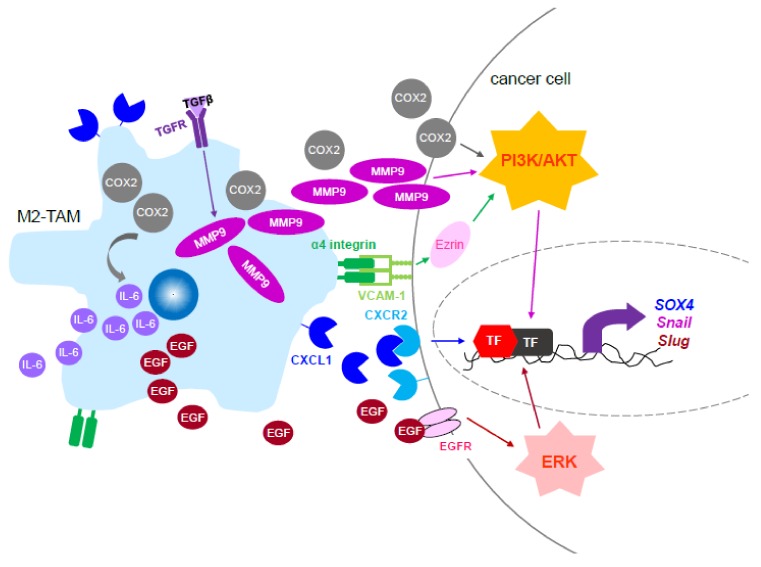
Signaling and the secreted factors of M2-TAM to promote cancer invasion and metastasis. The signaling molecules of M2-TAMs that modulate cancer cell migration, invasion, and metastasis through activation of phosphatidylinositol 3-kinase/human protein kinase B (PI3K/Akt) or ERK, either of which leads to upregulation of epithelial–mesenchymal transition (EMT) regulators, including Snail and Slug. Abbreviation, IL-6: interleukin-6; EGF: epithelial growth factor; EGFR: EGF receptor; MMP9: matrix metalloproteinase 9; VCAM-1: vascular cell adhesion protein-1; TF: transcription factor.

For example, IL-10 released by resident TAMs inhibits pathogen degradation by suppressing T helper cells and natural killer cells. It also allows the tumor to grow and divide without interference from immune cells that eliminate tumor cells [66]. Meanwhile, VEGF-A released by the M2d TAMs promoted outgrowth of endothelial cells to generate new blood vessels as a highway system for cancer cells to escape from the primary site [67]. Another common feature of the TME is hypoxia. A large body of evidence has demonstrated that several chemokines, including CCL2, CCL5, and CSF-1, are released under hypoxic conditions to promote the migration of TAMs into the nutrient-deprived region of cancer [68]. In addition, the M2d TAMs also secrete TNF to promote tumor cell glycolysis, thus providing additional oxygen to the hypoxic tumor cell and further contributing to cancer dissemination [69]. Furthermore, TAMs also promote cancer invasion through altering various signaling pathways in cancer cells (Figure 2). 

In breast cancer, it has been shown that the noncanonical Wnt 5a signaling activation in TAM promoted cancer cell migration through phosphorylation of JNK, followed by an increase in transcription of AP-1/c-Jun and matrix metalloprotease, MMP7, ultimately leads to the secretion of TNF [70]. Meanwhile, other chemokines, such as CXCL1, have also been demonstrated to enhance breast cancer migration, invasion, and EMT by upregulating the NF-KB/SOX4 signaling pathway [71]. CXCL1 was shown to bind to the SOX4 promoter and upregulated SOX4 transcription using the NF-κB pathway. In addition, knockdown of CXCL1 in THP-1-dervied macrophages suppressed breast cancer growth and lung metastasis in the orthotopic breast cancer xenografted mice. Another mechanism that TAMs utilize to regulate breast cancer metastasis to the lungs is through the expression of α4 integrins of TAM that bind to VCAM-1 expressed by cancer cells [72]. Upon binding of VCAM-1 of cancer cells, it activated PI3K/AKT signaling through the cytoplasmic peripheral protein Ezrin, leading to protection of the migrated breast cancer cells from potential cell death in the lungs. Intriguingly, another subset of macrophages that expressed VEGFR1 was enriched in the lung metastases of breast cancer [73]. In addition, these VEGFR1^+^ macrophages also regulated inflammatory genes to promote cancer seeding and growth in the lungs. In addition to supporting the pro-survival ability of metastatic breast cancer cells, aberrant PI3K/Akt signaling also participates in different mechanisms that lead to the potentiation of cancer metastasis. In the early stages of gastric cancer metastasis, MMP-9 secreted from TAMs induced EMT by upregulation of transcription factor Snail controlled by PI3K/AKT activation [74]. Besides MMP9, COX-2 was also released by TAMs to activate Akt signaling, which led to increased metastasis of breast cancer [75]. Furthermore, COX-2 in TAMs simultaneously induced the release of IL-6, a well-known metastatic promoting factor, from TAMs to enhance cancer metastasis. Another group demonstrated that activation of TLR4 signaling led to IL-10 production in M2-polarized macrophages promoting EMT of pancreatic cancer cells via its regulation on mesenchymal markers, vimentin, and Snail [41]. In addition, blockade of TLR4 through its specific siRNA or neutralizing antibody in the co-culture system of M2 TAM with pancreatic cancer cells abolished the TAM-increased mesenchymal markers in cancer cells. 

During tumor metastasis, cancer cells migrate to other parts of the body where proliferation is induced. To leave the original site, cancer cells have to break their physical contact with the basement membranes. A very common process for breaking containment of the basement membrane is by activation of MMPs. Accumulating evidence has shown that cancer cells activate various types of MMPs, including MMP2, MMP7, MMP9, and MMP14, to facilitate migration to other organs [76,77,78]. Besides cancer cells, stroma cells, especially TAMs, have been reported to regulate MMPs leading to elevated metastasis. TAMs of gastric cancer released MMP9, which promoted cancer cell migration through an induction of the master regulator of EMT, Snail [74]. TAMs in larynx carcinoma were also reported to secrete MMP9 to promote cancer growth and metastasis [79]. Furthermore, it was shown that MMP9 was downstream of TGFβ receptor signaling of TAMs, which were stimulated by cancer cells expressing placental growth factor (PLGF), suggesting that cancer cells were able to potentiate MMPs levels through both themselves and their nearby stroma cells, such as TAMs. Of note, TAMs that express high levels of TGFβ1 were at the invasive front of CD133^+^ glioma stem-like cells, which are responsible for glioma invasion [80]. In addition, neutralization of TGFβ1 in the co-culture of primary murine CD133^+^ glioma stem-like cells and TAMs attenuated the invasiveness of the CD133^+^ glioma stem-like cells. Intriguingly, TGFβ1 secreted by TAMs also increased expression of MMP9 in glioma stem-like cells that contributes to the invasive ability of these cancer stem cells. 

**Table 3:** Summary of TAM cytokines and their signaling targets that result in elevated cancer metastasis.

TAM	Cancer			Reference
*Cytokine*	*Signaling Pathway*	*Signaling Target*	*Type*	
Wnt 5a	JNK	Ap-1/c-Jun, MMP7	breast cancer	[70]
CXCL1	NF-κB	NF-κB, Sox4	breast cancer	[71]
intergrin α4	PI3K, Akt	ND *	breast cancer	[72]
MMP9	PI3K, Akt	snail	gastric cancer	[74]
COX-2	Akt	ND *	breast cancer	[75]
IL-10	ND	snail, vimentin	pancreatic cancer	[41]
MMP9	ND	ND *	larynx carcinoma	[79]
TGFβ1	ND	MMP9	glioma	[80]
EGF	ERK, IncRNA	N-cadherin, vimentin	ovarian cancer	[81]
ND **	ERK	slug	lung cancer	[82,83]

ND *: not determined in the original publication; ND **: not determined the original publications and only indicated the effect through co-culture of M2-TAM with cancer cells without identifying the TAM secreted cytokines involved in promoting cancer metastasis through the epithelial-mesenchymal transition process.

In addition to cytokines and chemokines, TAMs also secrete growth factors to augment cancer metastasis. It has been shown that M2-like TAMs in ovarian cancer produced EGF to potentiate cancer cell migration and metastasis [81]. The ovarian cancer cells stimulated by EGF of TAMs underwent EMT through upregulation of N-cadherin and vimentin, activation of EGFR/ERK signaling, and downregulation of IncRNA inhibiting metastasis (LIMT) expression. Moreover, manipulation of EGFR signaling by the use of an EGFR inhibitor AG1478 or LIMT through the overexpression system in ovarian cancer cells diminished cancer migration and metastasis that were mediated by TAMs in the co-culture system as well as in the xenografted mice. Similarly, while co-culturing with M2-TAMs, activation of ERK signaling was detected in lung cancer cells that transitioned from epithelial to mesenchymal cells by increased expression of the Slug transcription factor, leading to elevated invasion and metastasis of lung cancer [82,83].

## 4. Conclusion Remarks 

Macrophages play a critical role in maintaining a healthy body. They are the link between the innate and adaptive immune response, which fights foreign pathogens to prevent infection of the organism. Although macrophages are necessary and contribute to cellular homeostasis, they have also been indicated in tumor initiation, progression, and metastasis. The M2 subtype macrophages, also known as TAMs, are able to expedite tumor progression by altering the cellular microenvironment making it more conducive for supporting tumor growth. M2 macrophages release various cytokines, chemokines, and growth factors, which cause aberrant cell signaling, ultimately leading to the activation of various transcription factors that contribute to tumor initiation, progression as well as metastasis. The generation and secretion of these pro-cancerous factors in macrophages are enhanced by cancer cells, which, in turn, form a positive feedback loop to ensure unstoppable cancer growth. A comprehensive understanding of the interaction between tumor and macrophages, along with their signaling, will shed light on future cancer therapeutic strategies efficiently targeting both types of cells. 

## Figures and Tables

**Table 1:** Characteristics of M1 and M2 macrophages.

Macrophage Subtype.	M1	M2a	M2b	M2c	M2d
*Stimulation*	IFNγ, LPS, GM-CSF	IL-4, IL-13, fungal infection	IL-1R	IL-10, TGFβ, glucocorticoids	IL-6, leukocyte inhibitory factor, adenosine
*Receptor*	IFNγR, TLR4, CSF2Rα	TLR4, IL-4Rα	TLR4, IL-4Rα	TLR4, IL-4Rα	TLR4, IL-4Rα
*Signaling pathway*	JAK1/2, P38, MSK1/2	p38 MAPK, JAK1/2/3, PI3K	p38 MAPK, JAK1/2/3, PI3K	p38 MAPK, JAK1/2/3, PI3K	p38 MAPK, JAK1/2/3, PI3K
*Transcription Factors*	STAT1/2/5, AP1, IRF3/5, NF-κB	CREB, JMJD3, STAT6, IRF4, PPARγ	CREB, JMJD3, STAT6, IRF4, C/EBPβ	CREB, JMJD3, STAT6, IRF4, C/EBPβ	CREB, JMJD3, STAT6, IRF4, C/EBPβ
*Target Genes*	Nos2, Ciita, IL12b, inflammatory genes	Arg1, FIzz1, Ym1, CD206	Arg1, Fizz1, Ym1, CD206	Arg1, Fizz1, Ym1, CD206	Arg1, Fizz1, Ym1, CD206
*Cytokine Secretion*	TNF, IL-1, IL-6, IL-12, IL-23	IL-10, TGFβ, IL-1ra	IL-1, IL-6, IL-10, TNF	IL-10, TGFβ	IL-10, IL-12, TNF, TGFβ
*Chemokine Secretion.*	CCL10, CCL11, CCL5, CCL8, CCL9	CCL17, CCL22, CCL24	CCL1	CCR2	CCL5, CXCL10, CXCL16

**Table 2:** Summary of cytokines and their signaling targets leading to cancer initiation.

TAM	Tumor		Reference
*Cytokine*	*Signaling Target*	*Type*	
IL-6 *	STAT3, cyclin D1, c-myc	hepatocellular carcinoma	[30,31]
TNF, RANTES	NF-κB, MMP9	pancreatic ductal adenocarcinoma	[32]
SDF1	Akt, CXCR4	gliomas	[36]
CCL3, IL-1ra, osteopontin, M- CSF1, GDNF	Akt, CXCR4	prostate cancer	[37]
CCL4 *	STAT3, COX-2, c-myc, PTEN, p53	prostate cancer	[39]

*: the cytokines were secreted by monocytes instead of macrophages reported in the original publications.

## Abbreviations

ADM	acinar-to-ductal metaplasia
AR	androgen receptor
CSF-1	colony-stimulating factor 1
FPR2	formyl peptide receptor 2
GDNF	glial cell-derived neurotrophic factor
GPR18	G protein-coupled receptor 18
ECM	extracellular matrix
EGF	epithelial growth factor
EMR1	EGF-like module-containing mucin-like hormone receptor-like 1
EMT	epithelial–mesenchymal transition
ERK	extracellular signal-regulated kinase
GSK3β	glycogen synthase kinase 3β
HCC	hepatocellular carcinoma
ICAM-1	intracellular adhesion molecule 1
IFN-gamma	interferon gamma
IL-1 ra	interleukin 1 antagonist
IL-6	interleukin 6
JAK	Janus kinase
LIMT	IncRNA inhibiting metastasis
LPS	lipopolysaccharide
MCP-1	monocyte chemoattractant protein 1
MHC	major histocompatibility complex
MMP	matrix metalloprotease
NF-κB	nuclear factor kappa-light-chain-enhancer if activated B cells
NK	natural killer
PanIN	pancreatic intraepithelial neoplasia
PDAC	pancreatic ductal adenocarcinoma
PI3K	phosphatidylinositol 3-kinase
PIPNM3	phosphatidylinositol transfer protein membrane-associated 3
PLGF	placental growth factor
PTEN	phosphatase and tensin homolog
RANTES	regulated on activation, normal T cell expressed and secreted
STAT3	signal transducer and activator of transcription 3
TAM	tumor-associated macrophage
Treg	regulatory T
TGF-β	transforming growth factor β
TLR	toll-like receptor
TNF	tumor necrosis factor
VCAM-1	vascular cell adhesion protein 1
VEGF	vascular endothelial growth factor
VEGFR	vascular endothelial growth factor receptor
ZEB1	zinc finger E-box-binding homeobox 1
